# The role of salvianolic acid B and benzoylpaeoniflorin in enhancing angiogenesis through Nrf2/HO-1/VEGFA signaling axis in ischemic stroke recovery

**DOI:** 10.1080/13880209.2025.2605571

**Published:** 2025-12-23

**Authors:** Chao Zhao, Xiaodan Bai, Yi Ding, Aiguo Zeng, Aidong Wen, Qiang Fu, Jingwen Wang

**Affiliations:** ^a^ Department of Pharmacy, Xijing Hospital, Fourth Military Medical University, Xi’an, China; ^b^ Department of Pharmaceutical Analysis, School of Pharmacy, Xi’an Jiaotong University, Xi’an, China; ^c^ Department of Pharmacy, Tangdu Hospital, Fourth Military Medical University, Xi’an, China; ^d^ Department of Pharmaceutical Analysis, College of Pharmacy, Shenzhen Technology University, Shenzhen, China

**Keywords:** Salvianolic acid B, benzoylpaeoniflorin, ischemic stroke, angiogenesis, Nrf2/HO-1/VEGFA signaling axis

## Abstract

**Context:**

Angiogenesis is one of the essential protective mechanisms that promote neural repair and regeneration after ischemic stroke (IS). Salvianolic Acid B (SAB) and Benzoyl paeoniflorin (BP) are compounds extracted from the Chinese medicines *Salvia miltiorrhiza* Bunge and *Paeonia suffruticosa* Andrews, respectively.

**Objective:**

We investigated whether SAB combined with BP alleviated IS by promoting micrangium angiogenesis and determined the potential molecular mechanisms.

**Materials and methods:**

The impact of SAB-BP on angiogenesis after IS was investigated in middle cerebral artery occlusion (MCAO) rat model, ponatinib-induced ischemic stroke in zebrafish, and human umbilical vein endothelial cells (HUVECs). The neuroprotective effect of SAB-BP in rats was assessed using behavior tests and histopathological staining. The cerebral thrombosis assessment and angiogenesis assay were performed in the zebrafish model. Cell proliferation and angiogenesis in oxygen-glucose deprivation and reperfusion (OGD/R) HUVECs were assessed through cell viability, tube formation, migration, and invasion assays. Western blot analysis and immunofluorescence staining were used to determine the protein expression levels of Nrf2, HO-1, and VEGFA.

**Results:**

The findings indicated that SAB-BP significantly reduced neurological impairment following IS and promoted the formation of functional vessels in the cerebral ischemic penumbra. Furthermore, SAB-BP up-regulated the protein expression of Nrf2, HO-1, HIF-1α, and VEGFA. Intriguingly, the pro-angiogenic effect of SAB-BP markedly restrained by adding the inhibitor of Nrf2 (ML385).

**Discussion and conclusion:**

Our study demonstrates that SAB-BP enhances angiogenesis following IS by modulating the Nrf2/HO-1/VEGFA signaling axis both *in vivo* and *in vitro*. SAB-BP could serve as a promising therapeutic agent for IS recovery.

## Introduction

Ischemic stroke (IS), resulting from disrupted cerebral blood flow, causes major neural damage and is a leading global cause of death and disability (Caplan 1991). The management of IS has traditionally focused on the early reperfusion of at-risk tissue through intravenous thrombolysis and/or endovascular thrombectomy. However, surgical thrombectomy may paradoxically exacerbate ischemia-reperfusion injury, thereby worsening cerebral damage (Krishnan et al. 2021). Tissue plasminogen activator is the only FDA-approved drug for IS, but its use is limited by a short therapeutic window, reduced effectiveness, and potential for causing hemorrhagic complications. Increased microvessel density in the peri-infarct region indicates that angiogenesis regulation is vital for neural repair and regeneration after IS (Kanazawa et al. 2019; Fang et al. 2023).

The herbal formula of Salvia Miltiorrhiza (dried root of *Salvia miltiorrhiza* Bunge) combined with Cortex Moutan (dried root bark of *Paeonia suffruticosa* Andrews) has been extensively utilized in traditional Chinese medicine to treat cerebrovascular and cardiovascular diseases for many centuries in China (Wang et al. 2007; Li et al. 2018; Liu et al. 2023a). Salvianolic Acid B (SAB), a primary phenolic derivative from Salvia Miltiorrhiza (SM), demonstrates potential in mitigating cerebral I/R injury (Lv et al. 2015; Wang et al. 2016), anti-atherosclerosis (Zhao et al. 2023) and neuroprotection activities (Guo et al. 2022). Benzoylpaeoniflorin (BP) isolated from Cortex Moutan (CM) also exhibits anti-inflammatory and immunoregulatory activities (Zhang and Wei 2020; Kim et al. 2022). However, whether there is a synergistic effect of two active compounds isolated from SM-CM in IS is limitedly understood.

In response to inadequate oxygen supply, the brain initiates angiogenesis, a process that promotes blood vessel growth and supports neurological recovery following a stroke (Krupinski et al. 1994). Recent studies suggest that post-stroke angiogenesis in the penumbra, the at-risk but not yet infarcted tissue, can mitigate ischemic damage (Zhang et al. 2024). Angiogenesis serves as a crucial protective mechanism facilitating neural regeneration and functional recovery in stroke pathophysiology. Research has shown that SAB enhances angiogenesis in myocardial ischemia (Chen et al. 2023). However, whether SAB-BP can enhance cerebral angiogenesis to mitigate IS requires further investigation.

Notably, the Nrf2/HO-1/VEGFA axis plays a pivotal role in angiogenesis. Nrf2 is a crucial transcription factor involved in the regulation of oxidative stress. The Nrf2 pathway induces antioxidant proteins such as HO-1, quinone oxidoreductase-1, and glutathione, which exert vital neuroprotective effects in IS (Sun et al. 2023; Duan et al. 2024). Recent studies have highlighted the significant role of Nrf2 in angiogenesis. In human glioblastoma, the expression of Nrf2 can regulate angiogenesis in the tumor, and this regulation is mainly achieved by regulating the HIF-1α-VEGFA pathway (Ji et al. 2014). Kweider et al. found that in HUVECs, activation of downstream HO-1 through Nrf2-ARE can significantly increase VEGFA protein levels, thereby inducing angiogenesis (Kweider et al. 2015). Furthermore, studies have emphasized Nrf2’s crucial role in promoting angiogenesis by modulating VEGF (Li et al. 2016; Guo et al. 2018). However, no study reports the connection between SAB-BP and the Nrf2/HO-1/VEGFA axis in IS.

This article aimed to explore SAB-BP’s pro-angiogenic activity and mechanism in IS. *In vivo* studies, using rats in MCAO model for cerebral ischemia, revealed that SAB and BP synergistically alleviate brain injury and promote angiogenesis through phenotype-based efficacy screening. The pro-angiogenic effects were assessed by examining subintestinal vessels (SIVs) formation in transgenic zebrafish embryos. *In vitro*, SAB-BP-treated OGD/R-induced HUVECs were analyzed for tube formation and cell migration. In addition, transcriptomics in rat brain tissues was applied to forecast the targets and related pathways of SAB-BP in IS. Further analysis indicated their potential influence on gene expression within the Nrf2/HO-1/VEGFA axis.

## Materials and methods

### Cell lines and reagents

HUVECs were purchased from ScienCell Research Laboratories (Carlsbad, #8000, USA). Salvianolic Acid B (CAS: 115939-25-8, HS034208) and Benzoylpaeoniflorin (CAS: 38642-49-8, HB003022) were obtained from Baoji Herbest Bio-Technology Co., Ltd (Baoji, China), with purities exceeding 98% as determined by HPLC. Butylphthalide was provided by Shijiazhuang Pharmaceutical Group Co. Ltd (Shijiazhuang, China). Recombinant human VEGF protein (Cat. No. 100-20) were purchased from peprotech Bio-Technology Co., Ltd (Suzhou, China). Ponatinib (CAS: 943319-70-8, HY-12047) and ML385 (CAS: 846557-71-9, HY-100523) were purchased from MCE (NJ, USA). Trizol was purchased from Thermo Fisher Scientific (15596018CN, USA). Matrigel was acquired from Corning Incorporated (NY, USA).

### Experimental groups and establishment of MCAO model

This study involved ninety Sprague–Dawley rats, each weighing 220 to 240 grams, purchased from the Fourth Military Medical University in Xi’an, China (sourced from the Shanghai Model Organisms Center, Inc), license number for animal production: SCXK 2017-0021. All experimental procedures were approved by the Laboratory Animal Ethics Committee of the Fourth Military Medical University (Approval Nos: IACUC-202405077 and IACUC-202510003) and complied with NIH guidelines and ARRIVE guidelines. The rats were kept at 20–24 °C, 50–60% humidity, on a 12-hour light-dark cycle, with free access to food and water.

Rats were randomly assigned to six treatment groups: sham, MCAO, MCAO with SAB (20 mg/kg/day), MCAO with BP (20 mg/kg/day), MCAO with a SAB-BP combination (20 mg/kg/day), and MCAO with a positive control, Butylphthalide (NBP) at 5 mg/kg/day. The dosage regimens of SAB and BP for rats were determined according to our dose-finding experiments and previous studies (Lv et al. 2015; Wang et al. 2016; Zhou et al. 2020). The drugs were administered daily *via* intraperitoneal injection over a period of two weeks. The MCAO and sham groups received an equivalent volume of saline.

Adult rats underwent MCAO using the intraluminal filament method (Liao et al. 2023). Under 1% pentobarbital sodium (50 mg/kg) anesthesia, a filament was inserted into the external carotid artery and advanced 2 cm into the internal carotid artery to block the middle cerebral artery for 2 h, followed by reperfusion. Specifically, we measured regional CBF in rats’ ipsilateral cerebral cortex using a laser Doppler flowmetry (LDF) system (MoorVMS-LDF2, Moor Instruments, UK) at three time points: 10 min pre-filament insertion, 10 min post-insertion, and 10 min post-withdrawal. Valid MCAO models required occlusion-phase CBF ≤ 20% of baseline (effective occlusion) and reperfusion-phase CBF ≥ 50% of baseline (successful reperfusion). Animals failing these criteria were excluded. The sham surgery group underwent the procedure without filament insertion.

This study followed AVMA animal euthanasia guidelines to minimize pain and stress. Euthanasia was performed in rats exhibiting severe neurological deficits or infections *via* intraperitoneal injection of pentobarbital sodium at three times the anesthetic dose (150 mg/kg). The procedure lasted 2–3 min, with death confirmed by absence of heartbeat and respiration. The entire experiment spanned April to October 2024.

### Neurological functional analysis

Neurological function was assessed blindly on days 1, 3, 7, 10, and 14 post-MCAO surgery using the Zea-Longa (Liu et al. 2023b), rotarod test (Sunwoo et al. 2012) and corner test (Han et al. 2015) as previously described. Zea-Longa scores ranged from 0 (no issues) to 4 (no movement or consciousness). To evaluate forelimb and hindlimb coordination and balance, we used the rotarod test (LE8200 Panlab, USA). Rats experienced an accelerating rotor from 5 to 40 rpm over 300 s. A blinded investigator recorded how long each rat stayed on the rod (Xia et al. 2023). The corner test evaluates unilateral sensorimotor cortical impairment by placing rats between two 30° angled cardboard plates in their home cage. Post-ischemic and reperfusion injury, rats often turn toward the injured side, with right turns tracked over 10 trials.

### Brain infarct volume

After completing behavioral tests on day 14 post-MCAO, all rats were sacrificed. The brains were cut into five 2 mm slices, stained with 2% TTC, and incubated at 37 °C for 20 min in the dark. After fixing in 4% paraformaldehyde (Yu et al. 2023), photographs of the infarcted areas were taken and analyzed using Image J. Infarct percentage was calculated as (infarct volume/contralateral hemisphere volume) × 100%.

### H&E staining

According to the established experimental method of H&E staining, the brain tissues were fixed, embedded, and sectioned into 5 μm slides (Zhao et al. 2024a). Sections were immersed in Xylene I and II (20 min each), followed by 100% ethanol and 75% ethanol (5 min each). Post-dewaxing, they were stained with hematoxylin and eosin (3 min each), then mounted with neutral mounting medium.

### Nissl staining

After deparaffinization, ethanol dehydration, and deionized water rinsing, sections were immersed in 0.1% cresyl violet solution for 5 min, followed by 95% ethanol until Nissl bodies appeared dark blue. They were then dehydrated in 100% ethanol (5 min), cleared in xylene (5 min), and finally mounted with neutral mounting medium. Nissl bodies in cortical neurons were observed under a microscope, and images were captured and analyzed using Image J software (Song et al. 2021).

### TUNEL staining

TUNEL staining was conducted following established protocols utilizing recombinant terminal deoxynucleotidyl transferase (Fang et al. 2021). Neuronal apoptosis levels were assessed by counting TUNEL-positive cells in stained tissue sections using a fluorescence microscope, and images were analyzed using Image J.

### Immunohistochemical staining

The brain sections were prepared as mentioned above. Sections underwent conventional dewaxing, antigen retrieval, and PBS washes. Washed sections were incubated in 3% H_2_O_2_ (RT, 25 min, dark), then blocked with 3% BSA (RT, 1 h). Primary antibodies were incubated at 4 °C overnight, followed by secondary antibodies (RT, 1 h) and DAB chromogenic reaction. After nuclear counterstaining, dehydration, and mounting, the sections were observed and imaged microscopically. The antibody information is provided in Supplementary Table 1.

### Transcriptomic analyses

To investigate the molecular mechanisms of SAB-BP in improving rat stroke outcomes and promoting neovascularization. We conducted transcriptome analysis on ischemic penumbra brain tissues from rats in the sham group, MCAO group, and SAB-BP treatment group, 14 days following MCAO surgery. Following the Trizol-based extraction protocol, total RNA samples were quantified for concentration using a Nanodrop 2000 instrument (Thermo Scientific, USA) and subsequently analyzed for quality control *via* agarose gel electrophoresis. Following reverse transcription of mRNA into cDNA, libraries were constructed and sequenced on an Illumina Novaseq^™^ 6000 system by Gene Denovo Biotechnology Co., Ltd (Guangzhou, China) with 2 × 150 bp paired-end configuration. Raw reads were quality-controlled using FastQC (v0.11.9), and low-quality data were filtered out to obtain clean reads (parameters: Phred score ≥ 20, read length ≥ 50 bp). Bowtie2 (v2.5.3) and HISAT2 (v2.2.1) were employed to align transcriptomic reads to the reference genome. Statistically significant differentially expressed genes (DEGs) were identified through DEGseqR (v1.34.0) analysis (|log2 Fold Change| >1, and q-value < 0.05), followed by functional annotation using GO enrichment (http://www.geneontology.org/) and KEGG pathway analyses (https://www.genome.jp/kegg/) to elucidate SAB-BP’s molecular mechanisms in IS.

### Ethical considerations in zebrafish husbandry

Zebrafish’s small size and transparent larvae make it ideal for pharmacological phenotype analysis. In our study, we employed ponatinib to induce and establish a zebrafish model of ischemic stroke, aiming to evaluate the therapeutic efficacy of SAB and BP. As a BCR-ABL tyrosine kinase inhibitor, ponatinib elicits angiogenic defects, thrombosis, and reduced blood flow by impairing endothelial cell viability and proliferation, thereby suppressing the formation of subintestinal vessels (SIVs) and intersegmental vessels (ISVs) (Ai et al. 2018; Chen et al. 2022; Lin et al. 2023). This study utilized two zebrafish lines: *Tg (fli-1 EGFP)* for neovascularization assays and the Albino strain for other experiments. Zebrafish (license number for animal production: SCXK 2022-0004) were obtained from the Laboratory Animal Center of Hunter Biotech (Hangzhou, China). Zebrafish were housed in a tank at 28 °C. The water had a pH range of 6.5 to 8.5, salinity of 200 mg/L, conductivity between 450 and 550 μs/cm, and hardness of 50 to 100 mg/L CaCO_3_. All experiments with zebrafish were performed following the Laboratory Animal Center’s IACUC guidelines (Approval No. IACUC-2024-9280-01), NIH guidelines and ARRIVE guidelines. The zebrafish larvae were placed in a 0.016% Tricaine methane sulfonate (MS-222) working solution for 1–2 min. The experimental operation can be performed when the larvae stop swimming or show no response to touch. Regarding the euthanasia procedure, an overdose of tricaine methane sulfonate (1%) was used to ensure a humane end.

### Minimum toxic concentration (MTC) assay

510 two days post-fertilization (dpf) zebrafish were exposed to the test drug for 24 h, after which mortality and toxicity were assessed. They were first exposed to 1 μg/mL ponatinib for 24 h, then treated with different concentrations of SAB or BP (31.2, 62.5, 125, 250, and 500 μg/mL) or combined SAB-BP concentrations (15.6 + 15.6, 31.25 + 31.25, 62.5 + 62.5, 125 + 125, 250 + 250 μg/mL) for another 24 h. MTC was identified as the highest concentration that did not produce observable adverse effects in zebrafish. Dead larvae were removed and recorded to maintain water quality, while all larval groups were kept at 28.0 °C.

### Cerebral thrombosis assessment

180 two dpf zebrafish embryos were placed in 6-well plates with 30 embryos per well. DMSO (0.1%) served as the blank control, while ponatinib (1 μg/mL) caused angiogenic defects in the model group. Treatment groups were co-treated with ponatinib and various drugs, including SAB, BP, SAB-BP, and Edaravone (positive control, 5 μg/mL). The drug dosages were as follows: SAB at 125 μg/mL, BP at 125 μg/mL, and SAB-BP with each component at 62.5 μg/mL. After a 24-hour drug exposure, o-dianisidine staining was applied for 15 min in darkness. Zebrafish were examined and photographed using an OLYMPUS SZX7 microscope to identify and count cerebral thrombosis cases, facilitating the calculation of thrombus incidence. Quantitative analysis of cerebral thrombosis areas was conducted using NIS-Elements software (D3.10 201, Nikon, Japan).

### Neovascularization assessment

We selected 60 normally developing 3 dpf *Tg (fli-1: EGFP)* transgenic zebrafish for treatment. Zebrafish exposed with ponatinib (1 μg/ml) for 24 h as the model group. Drug treatment was administered as described in ‘cerebral thrombosis assessment’. After 24 h, a fluorescence microscope (AXIOZoom.V16, ZEISS, Germany) was used to visualize and enumerate the number of subintestinal vessels (SIVs) trunks and sprouting branch vessels formed (the ‘angiogenic basket’ area marked by yellow dashed lines). Subsequent data analysis was performed using NIS-Elements software (Nikon, Tokyo, Japan). Quantitative results were presented as ‘the number of SIV sprouts per zebrafish larva (mean ± SD)’.

### OGD/R model and cell treatment approaches

Cells were first cultured in a serum-free, glucose-free medium under hypoxia (0.3% O_2_, 5% CO_2_, and 94.7% N_2_) for 4 h, then incubated in a complete medium under normoxia for 24 h (Zhu et al. 2022). The experimental groups included control, OGD/R, SAB + OGD/R (20 μM), BP + OGD/R (16 μM), SAB-BP + OGD/R (10 μM, 8 μM), SAB-BP-ML385 + OGD/R (10 μM, 8 μM, 1.9 μM), and VEGFA + OGD/R (10 ng/ml). ML385 was employed to selectively inhibit Nrf2 activity in cells. HUVECs maintained under standard conditions served as the control group. The HUVECs in the treatment groups underwent OGD/R simultaneously with drugs or VEGFA treatment.

### Cell viability assay

The CCK8 assay (GLPBIO, #GK10001) was used to assess cell viability. Following OGD exposure and 24-hour treatment preincubation, CCK8 solution was added and incubated for 2 h at 37 °C. OD at 450 nm was then measured with a Multiscan MK3 microplate reader.

### Tube formation analysis

In the tube formation analysis, matrigel was thawed overnight, solidified on a pre-cooled culture plate at 37 °C for 2 h, and HUVECs were seeded at 2 × 10^4^ cells per well on 24-well plates. After 4–6 h of incubation, images were captured using an OLYMPUS CKX41 inverted fluorescence microscope, and Image J analyzed the total tube length and connection points.

### Cell migration assay

Transwell inserts (REF353097, BD, USA) were used to evaluate the migration of HUVECs. After resuspension in a serum-free medium, cells were added to transwell inserts, with each well containing roughly 1 × 10^5^ cells. They migrated through the 8.0 µm pore polyethylene terephthalate membrane to the underside containing complete medium. Cells were treated with different drugs and incubated for 24 h at 37 °C with 5% CO_2_. They were then extracted using cotton swabs, stained with crystal violet, and quantified in three random microscopic fields.

### Cell wound scratch assay

Scratch assays evaluated cell motility using 6-well plates marked with horizontal lines. HUVECs in their growth phase were cultured at 37 °C with 5% CO_2_. The following day, a vertical scratch was made, and after washing with PBS, cells were incubated for 24 h in serum-free ECM with different drug concentrations. Migration was imaged at the beginning and end of incubation using an inverted microscope.

### Western blot assay

Using the standard method (Li et al. 2025b), brain tissues and HUVECs were lysed with RIPA buffer to extract proteins and measure their concentration. Proteins underwent gel electrophoresis, were transferred to a PVDF membrane, blocked with 5% BSA, and incubated overnight with a primary antibody at 4 °C. After washing, the secondary antibody was applied for 2 h, and then protein bands were then detected and quantified with Image J software. The antibody information is provided in Supplementary Table 1.

### Immunofluorescence assay

Following the method described earlier, immunofluorescence was performed (Song et al. 2022). After the animals were perfused, the brain tissues were fixed for 24 h. Brain tissues were cut into 4 µm sections, deparaffinized, rehydrated, and subjected to heat-induced epitope retrieval. They were then blocked with donkey serum, and primary antibodies were incubated overnight at 4 °C. For HUVEC samples, fixation and blocking used paraformaldehyde and horse serum, respectively, with similar antibody incubation. Both sections and cells were washed, stained with secondary antibodies and DAPI, and mounted with antifade medium. Specimens were examined under a fluorescence microscope, and images were analyzed using Image J. For vessel quantification, a CD31-positive vessel separated from adjacent ones was counted as one. Immunofluorescence results were expressed as the mean number of positive cells/vessels per field. The antibody information is provided in Supplementary Table 1.

### Quantitative real-time PCR assay

Previously extracted brain tissue RNA was reverse transcribed into cDNA using HiScript II RT SuperMix (Vazyme Biotech, China). Quantitative real time PCR was then performed with a Veriti 96-Well Thermal Cycler (Applied Biosystems, USA). Using cDNA as template, qPCR was run on the StepOnePlus Real-Time PCR System (Applied Biosystems, USA) with ChamQ SYBR Color qPCR Master Mix (Vazyme Biotech, China). All procedures followed manufacturers’ instructions. Each reaction included 3 replicates for selected genes. Relative gene expression was calculated *via* the 2^−ΔΔCT^ method, using β-actin as the reference gene and cycle thresholds of target and reference genes. Primer sequences are listed in Supplementary Table 2.

### Statistical analysis

Results were presented as mean ± SD analyzed with the aid of GraphPad Prism 10.0. An unpaired two-tailed Student’s t-test assessed differences between two groups, while one-way ANOVA was used for multiple groups. Two-way repeated measures ANOVA with Bonferroni post hoc tests compared treatment groups over time in the Zea-Longa, rotarod, and corner tests. Separately, the incidence of cerebral thrombosis in zebrafish was analyzed using the chi-square test. Differentially expressed genes were analyzed using DESeq and DESeq2, with a *p*-value < 0.05 indicating significance.

## Results

### SAB-BP treatment mitigated the neurobehavioral deficits in MCAO rats

**Figure 1.**
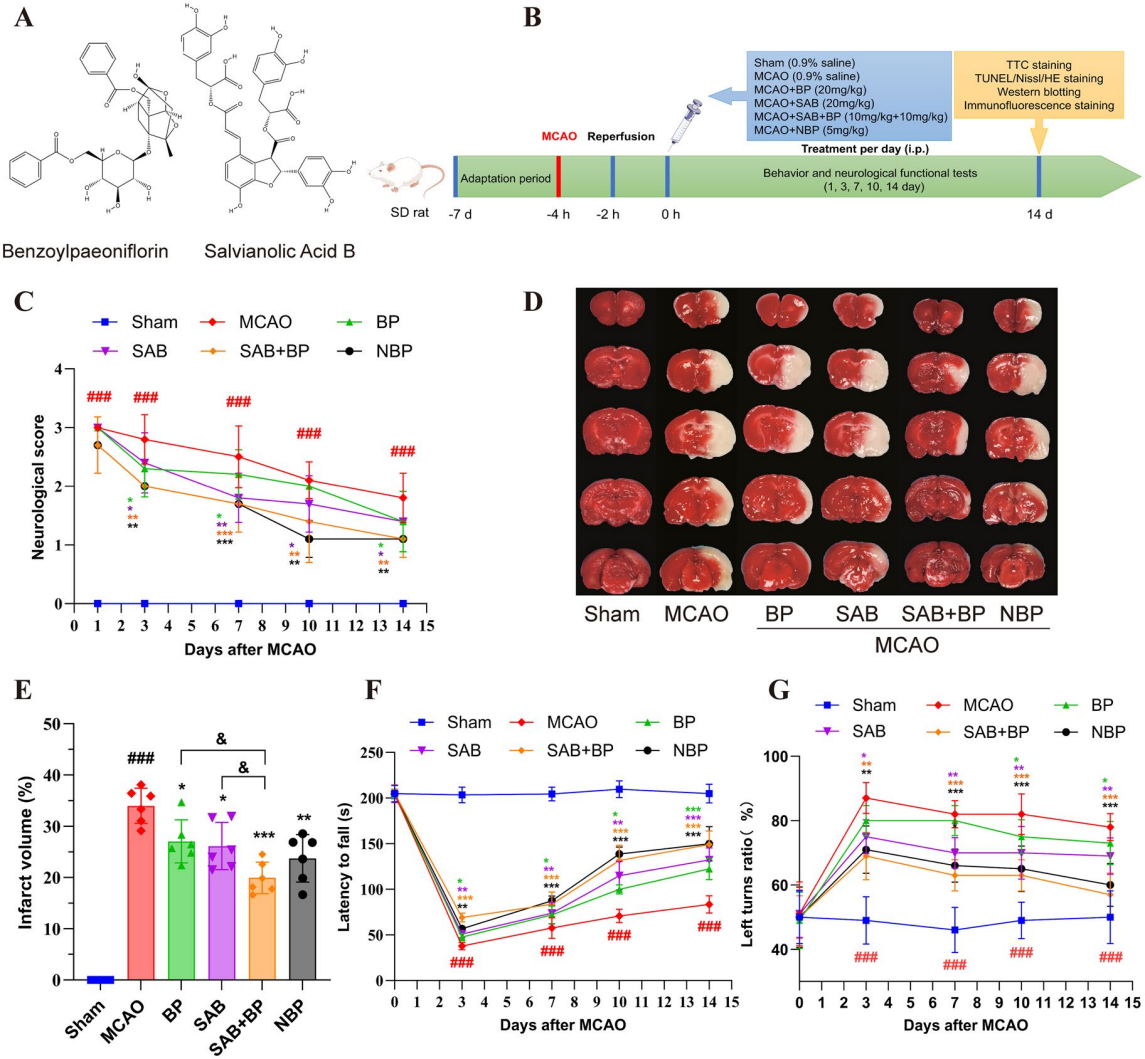
SAB-BP enhances neurological function in MCAO rats (*n* = 10). (A) the structure of compound SAB and BP. (B) Diagrammatic representation of the protocol used in animal experiments. (C) Neurological scores were evaluated at 24 h after reperfusion to 14 days. (D, E) TTC staining revealed infarct size in entire rat brain tissues post-MCAO. Rotarod test (F), and corner test (G) in MCAO rats. Data was expressed as mean ± SD (*p < 0.05, **p < 0.01, and ***p < 0.001 vs. MCAO group; ### *p* < 0.001 vs. sham group; & *p* < 0.05 vs. SAB+BP group).

The molecular structures of SAB and BP were shown in Figure 1(A). Neurological function, histochemical staining and immunological detection were assessed in rats following MCAO (Figure 1(B)). Neurological scores were elevated 24 h post-MCAO, confirming the successful establishment of the model. Drug treatment groups demonstrated improved neurological scores from 24 h to 14 days compared to the MCAO group (Figure 1(C)). The treatment groups could significantly reduce the volume of cerebral infarction. Both SAB and BP, when administered individually, significantly reduced infarct size, although their effects were less pronounced than those observed with the combined SAB-BP treatment (*p* < 0.05), as shown in Figure 1(D,E). The locomotor capabilities of the animals subjected to experiments were evaluated through the rotarod and corner assessments. In the rotarod test (Figure 1(F)), animals in the MCAO group showed impaired motor coordination, as evidenced by a reduced duration of time spent on the rotarod. Following drug administration, the duration that SAB-BP group animals stayed on the rotarod increased (*p* < 0.001). In the corner test (Figure 1(G)), animals in the MCAO group demonstrated a greater frequency of right turns in comparison to the sham group. The treatment with SAB-BP led to a decrease in the tendency for right turns among MCAO animals (*p* < 0.01). These results indicate that SAB-BP is more effective in promoting the recovery of motor, balance, and reflex functions in animals with abnormalities induced by MCAO than SAB or BP when used separately.

### Apoptosis, pathological alterations, and angiogenesis levels in rat cortex

**Figure 2.**
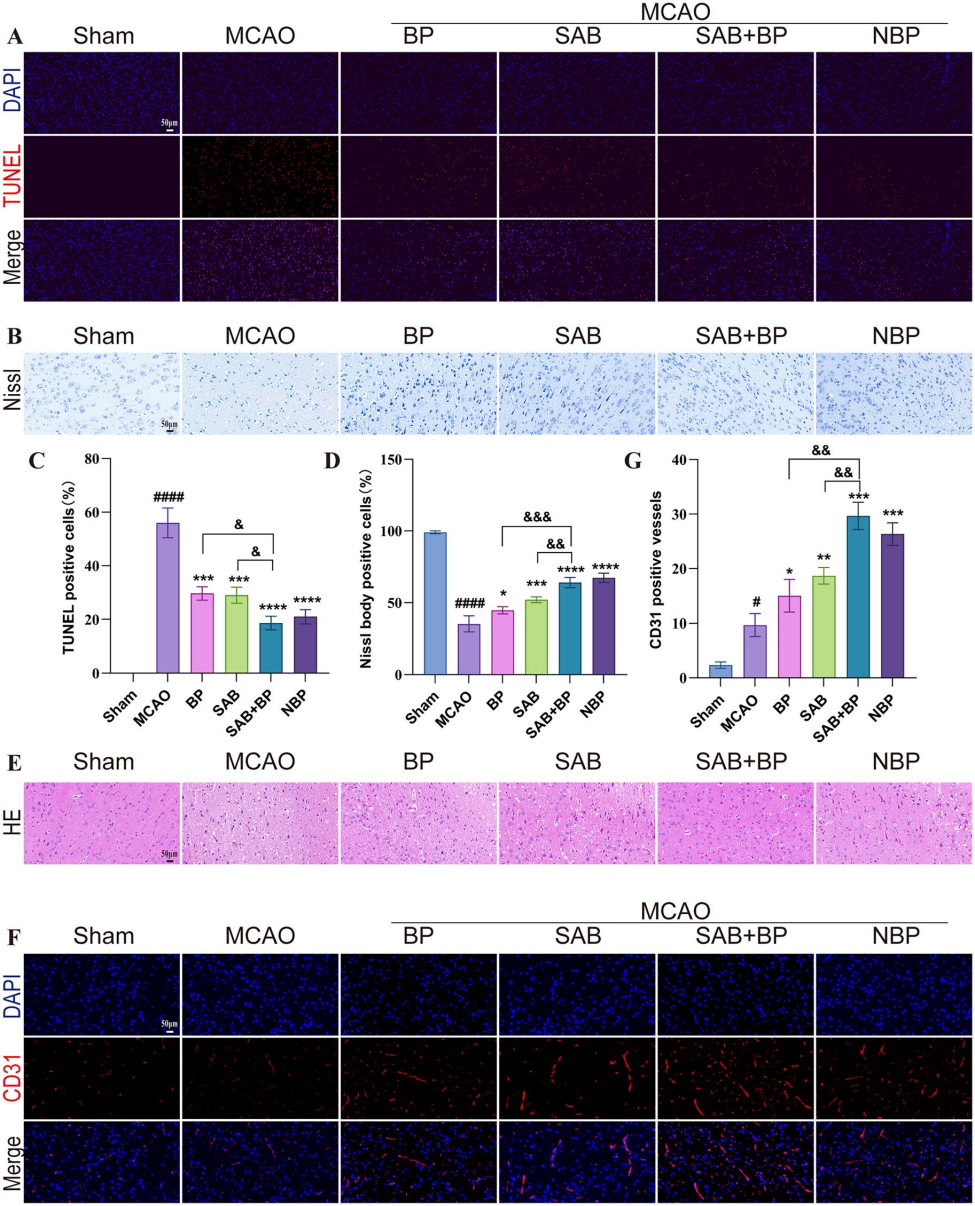
Impact of SAB-BP on apoptosis, pathology, and angiogenesis following MCAO in rat brains (*n* = 3). Microscopic images and statistical analysis of rat brain tissues include TUNEL staining (A and C), Nissl staining (B and D), and HE staining (E), with a scale bar of 50 μm. (F and G) Angiogenesis was evaluated by immunofluorescence staining of CD31 (scale bar = 50 μm). Data was expressed as mean ± SD (*p < 0.05, **p < 0.01, ***p < 0.001, and ****p < 0.0001 vs. MCAO group; #*p* < 0.05, ####*p* < 0.0001 vs. sham group; ^&^*p* < 0.05, ^&&^*p* < 0.01, and ^&&&^*p* < 0.001 vs. SAB+BP group).

The research assessed SAB-BP’s protective impact on brain cell damage after MCAO, with apoptosis detected through TUNEL staining. In the MCAO group, a substantial presence of apoptotic bodies was observed (with a positive rate of 56%). The level of cell apoptosis in the BP, SAB, and SAB-BP groups were significantly reduced, with positive rates of 30%, 29%, and 19%, respectively. Notably, SAB-BP group exhibited a markedly lower number of apoptotic cells compared to the individual treatment groups of SAB or BP alone, as shown in Figure 2(A,C). Neuron nucleosomes are crucial components linked to neuronal function. Nissl staining revealed that the MCAO group had a Nissl body positive rate of only 35% (vs. sham group), along with significantly reduced neuronal volume and widened intercellular gaps. Following treatment with BP, SAB, and SAB-BP, the nissl body positive rate increased to 45%, 52%, and 64%, respectively, with concurrent improvements in cell morphology. Notably, as shown in Figure 2(B,D), the number of Nissl bodies in the SAB-BP group was significantly higher than that in the treatment groups using SAB or BP alone. Moreover, H&E staining in Figure 2(E) showed that most neurons in the cerebral cortex ischemic penumbra of MCAO rats displayed swelling and nuclear pyknosis. The drug treatment group exhibited reduced cortical neuronal cell vacuolization and normalized cell morphology. The findings indicate that SAB-BP mitigates cerebral ischemia damage. To investigate the effects of combination treatment on angiogenesis, immunofluorescence staining of cerebral microvessels was performed using CD31, a marker for brain endothelial cells. Following 14 days of drug treatment, CD31- positive vessel density showed a significant increase relative to the MCAO group. The SAB-BP group showed a significant increase in CD31 positive vessel density in the perilesional cortex compared to the monotherapy group (Figure 2(F,G)). However, angiogenesis after stroke can be maladaptive if vessels are immature, leaky, or inflammatory in nature (Rust 2020). To investigate SAB-BP’s effect on BBB maintenance, we performed immunohistochemical staining for claudin-5 and ZO-1. Results showed each treatment group significantly upregulated claudin-5 and ZO-1 vs. the MCAO group. Additionally, their expression in the SAB-BP group was significantly higher than in the single-drug groups (Supplementary Figure 1).

### Transcriptome profile of SAB-BP promoting angiogenesis in rats

**Figure 3.**
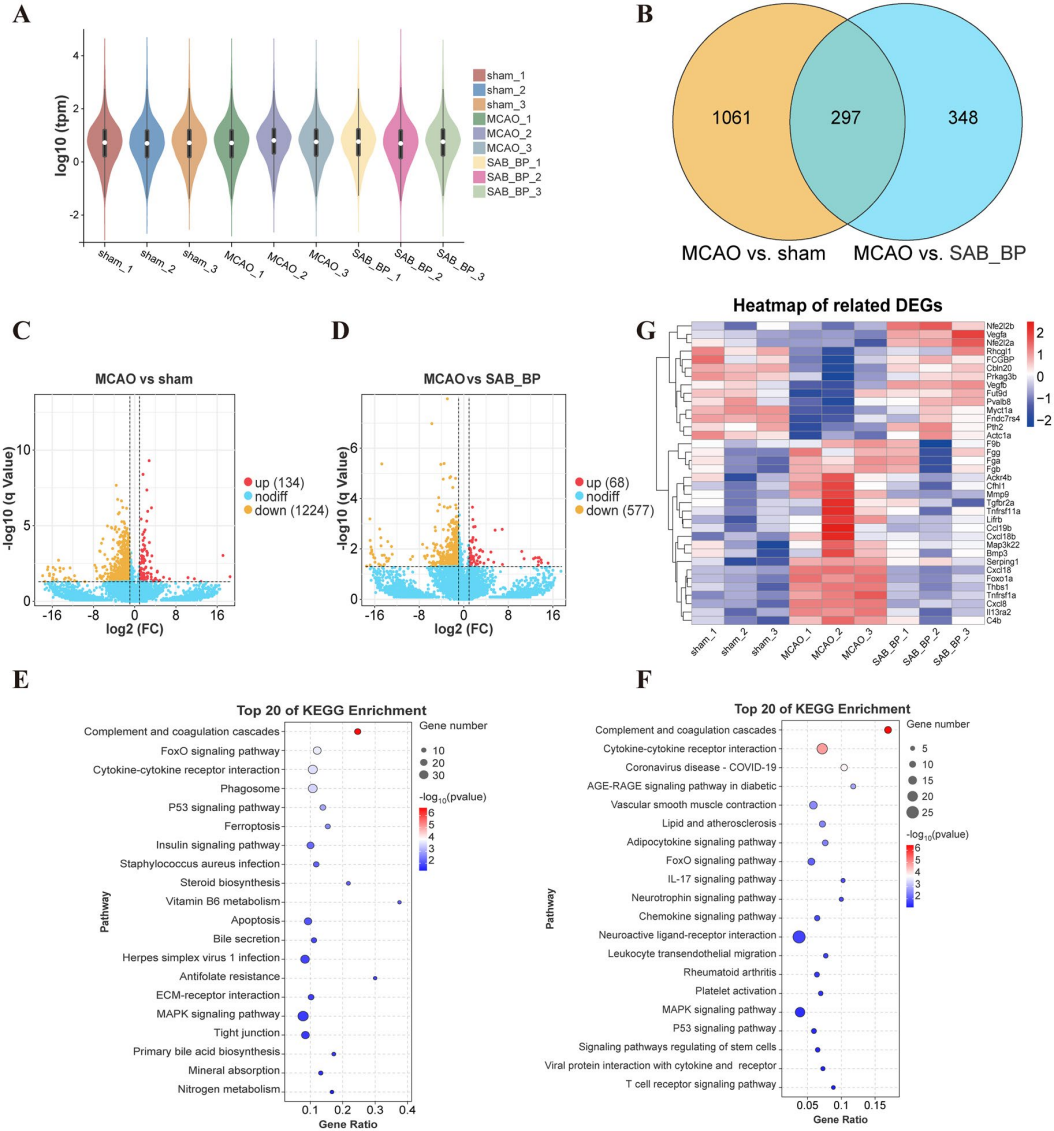
Transcriptomics analysis of SAB-BP treated rats MCAO model. (A) Violin plot illustrating TPM distribution across groups. (B) The Venn diagram displayed the overlapping DEGs. (C, D) DEGs volcano map of MCAO vs. sham, and MCAO vs. SAB-BP. (E, F) KEGG pathway annotation and enrichment analysis for MCAO vs. sham, and MCAO vs. SAB-BP. (G) Heatmap for hierarchical cluster analysis of DEGs between the samples.

We conducted transcriptome analysis on rats from the sham, MCAO, and SAB-BP groups to explore the mechanisms behind SAB-BP’s anti-cerebral ischemic and pro-angiogenic effects. Figure 3(A) demonstrates that the gene expression patterns were largely uniform across the three groups. Following this, the study analyzed the DEGs among the groups. The MCAO group exhibited 134 upregulated and 1224 downregulated DEGs compared to the sham group. In contrast to the SAB-BP group, the MCAO group exhibited 68 DEGs that were upregulated and 577 downregulated (Figure 3(C,D)). The three groups underwent a comprehensive analysis, resulting in the identification of 297 overlapping DEGs, visually represented using a Venn diagram (Figure 3(B)). Subsequently, KEGG enrichment analysis was conducted on DEGs, and the top twenty pathways with the most significant differences were selected based on Log P values. The DEGs between the MCAO and sham groups were associated with cytokine-cytokine receptor interaction, FoxO, P53, complement-coagulation cascades, and MAPK signaling pathways (Figure 3(E)). In the MCAO vs. SAB-BP groups, KEGG enrichment analysis identified potential anti-ischemia targets within the complement-coagulation cascades, the FoxO, IL-17, cytokine-cytokine receptor interaction, and MAPK signaling pathways (Figure 3(F)). The cytokine-cytokine receptor interaction and complement-coagulation cascades were identified as the primary enriched pathways through which SAB-BP mediates its anti-ischemic effects. Figure 3(G) shows a heatmap after additional cluster analysis of the enriched pathways related genes, which demonstrated that SAB-BP could significantly up-regulate the expression of VEGF, Nrf2 related genes and down-regulate the expression of Fibrinogen, Cxcl18, TNF and MAPK related genes. We quantified the mRNA expression levels of the aforementioned key genes using qRT-PCR. The results revealed that SAB-BP significantly upregulated the mRNA expression of Nfe2l2, Vegfa, and Vegfb, while notably downregulating that of Mmp9, Thbs1, Serping1, and Tnfrs11a (Supplementary Figure 2).

### *SAB-BP promoted angiogenesis via the Nrf2/HO-1 and HIF-1*α*/VEGF pathways*

**Figure 4.**
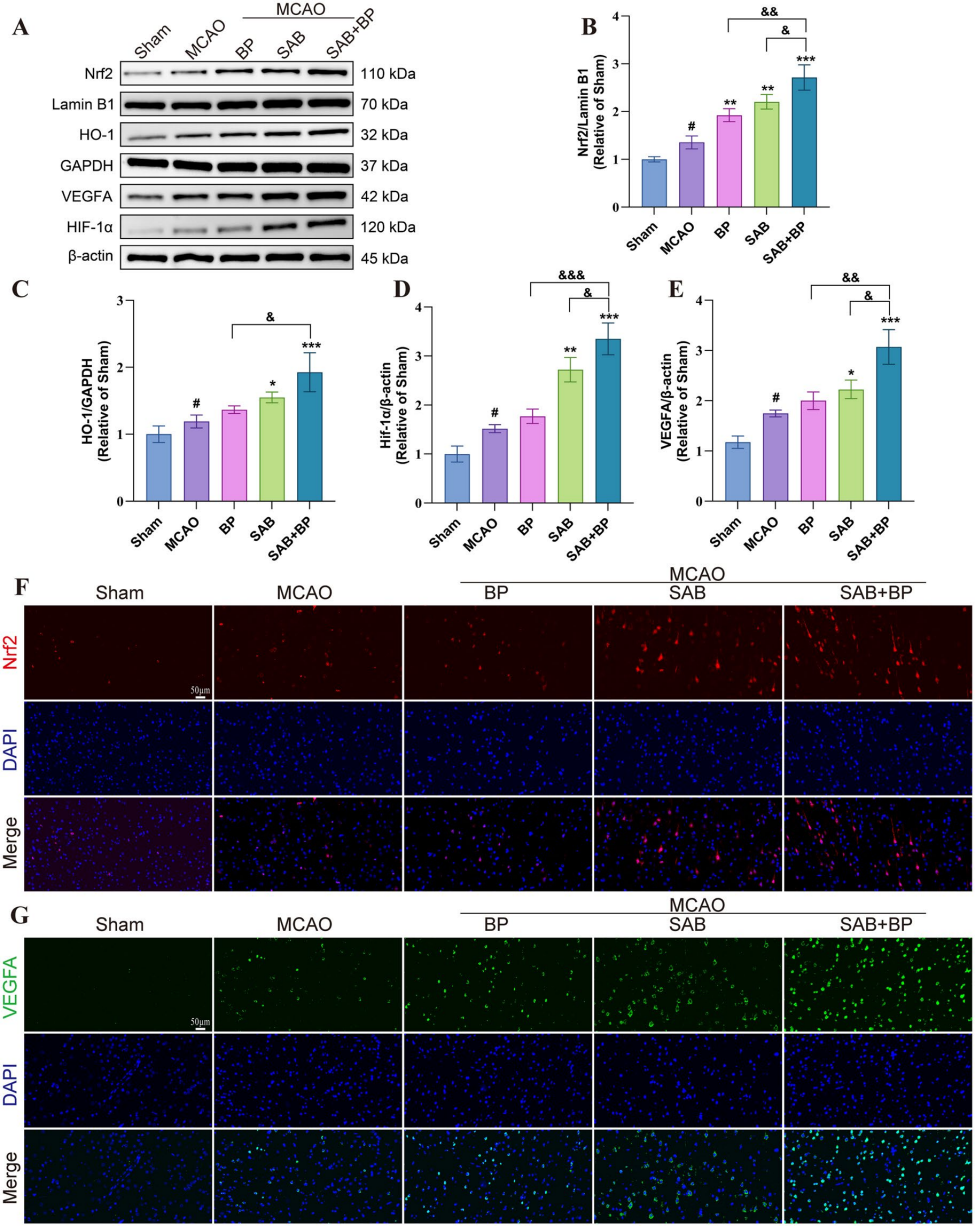
Effects of SAB-BP on key signaling protein expression in ischemic brain tissue (*n* = 3). (A) Western blot images depict the expression levels of Nrf2, OH-1, VEGFA, and HIF-1α. (B–E) Quantitative evaluation of Nrf2/Lamin B1, HO-1, VEGFA, and HIF-1α. Data was expressed as mean ± SD (*p < 0.05, **p < 0.01, and ***p < 0.001 vs. MCAO group; #*p* < 0.05, ##*p* < 0.01 vs. sham group; & *p* < 0.05, && *p* < 0.01, and &&& *p* < 0.001 vs. SAB+BP group). (F, G) The immunofluorescence staining results for Nrf2 and VEGFA are shown in detail (scale bar = 50 μm).

The key mechanism in the treatment of IS involves anti-oxidative stress and pro-angiogenesis following IS. The protein expression of Nrf2, HO-1, VEGFA, and HIF-1α were significantly upregulated in the drug treatment groups compared to the MCAO group (*p* < 0.001), as illustrated in Figure 4(A–E). In addition, protein expression significantly increased in the SAB-BP group when compared with the monotherapy group. Immunofluorescence studies, detailed in Figure 4(F,G), corroborated these findings, demonstrating the superior efficacy of the SAB-BP group over monotherapy in upregulating Nrf2 and VEGFA expression.

### SAB-BP promoted angiogenesis in the zebrafish model

**Figure 5.**
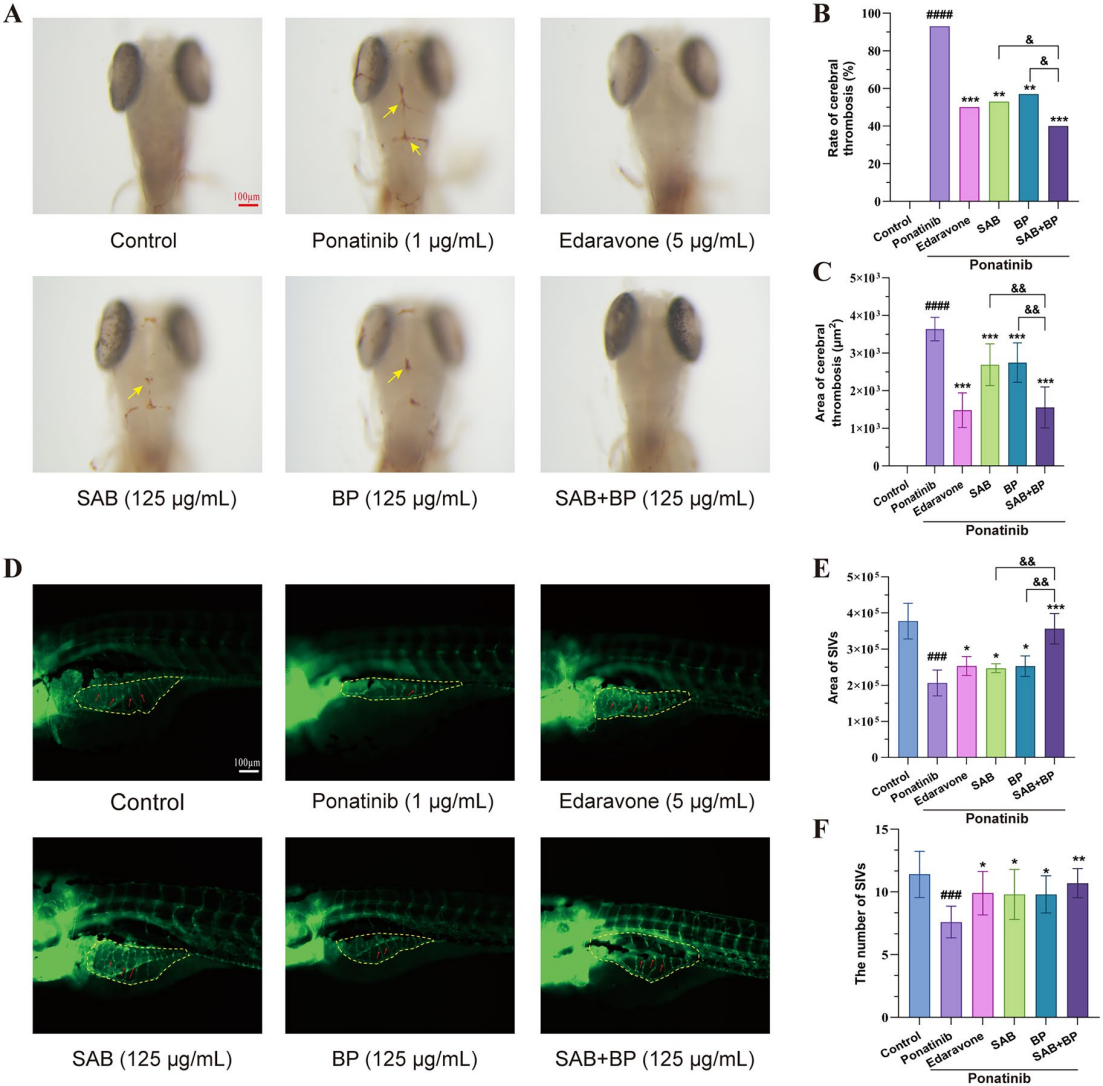
SAB-BP treatment promoted the vessel formation in zebrafish (*n* = 10). (A) Alterations in cerebral thrombosis at 3dpf in zebrafish larvae subjected to SAB, BP, and ponatinib exposure. (B) The rate of cerebral thrombosis of the different treatment groups. (C) The cerebral thrombosis regions across various treatment groups. (D) Fluorescent microscopy imaging of *Tg (fli-1: EGFP)* embryos at 4 dpf treated with DMSO or different drugs (scale bar = 100 μm). (E, F) Total SIVs count and area measurements in the zebrafish angiogenesis assay. Statistical significance: *p < 0.05, **p < 0.01, ***p < 0.001 vs. ponatinib group; #*p* < 0.05, ##*p* < 0.01, and ###*p* < 0.001 vs. control group. & *p* < 0.05, && *p* < 0.01 vs. SAB+BP group.

Initially, we investigated the toxic effects of SAB or BP on zebrafish. Zebrafish treated with 125 μg/mL SAB or BP did not exhibit morphological malformations. Consequently, 125 μg/mL was selected as the maximum concentration for zebrafish experiments. SAB and BP were mixed in a 1:1 ratio, and the MTC of SAB-BP was determined to be 62.5 + 62.5 μg/mL. Figure 5(A) presents images of zebrafish larvae’ brains stained with o-dianisidine. Compared with the control group, ponatinib induced cerebral thrombosis from 2dpf to 3dpf. In contrast, treatment with SAB or BP significantly reduced cerebral thrombosis, with the most marked reduction observed in SAB-BP-treated embryos. As shown in Figure 5(B,C), statistical analyses revealed that both the incidence and area of thrombosis in the SAB-BP combination therapy group were significantly lower than those in the monotherapy groups. The *Tg (fli-1:EGFP)* zebrafish model was utilized for *in vivo* angiogenesis research. The sprouting of subintestinal vessels (SIVs) in zebrafish, a key angiogenesis process, was utilized in our study to assess the effects of SAB, BP, and their admixture. Confocal microscopy was used to visualize blood vessels in live zebrafish with fluorescently labeled endothelial cells at 72 h post-fertilization. As anticipated, the number and area of SIVs were significantly higher in the SAB, BP monotherapy group and SAB-BP group than the model group (*p* < 0.0001), as shown in Figure 5(D) (the yellow dashed line demarcates the SIV area; red arrows highlight representative SIVs). The quantitative analysis of zebrafish SIVs area and number indicated SAB-BP treatment promoted angiogenesis better than the single component in zebrafish as shown in Figure 5(E,F), which indicates a proangiogenic effect of SAB-BP on zebrafish.

### SAB-BP enhances angiogenic functions in HUVECs

**Figure 6.**
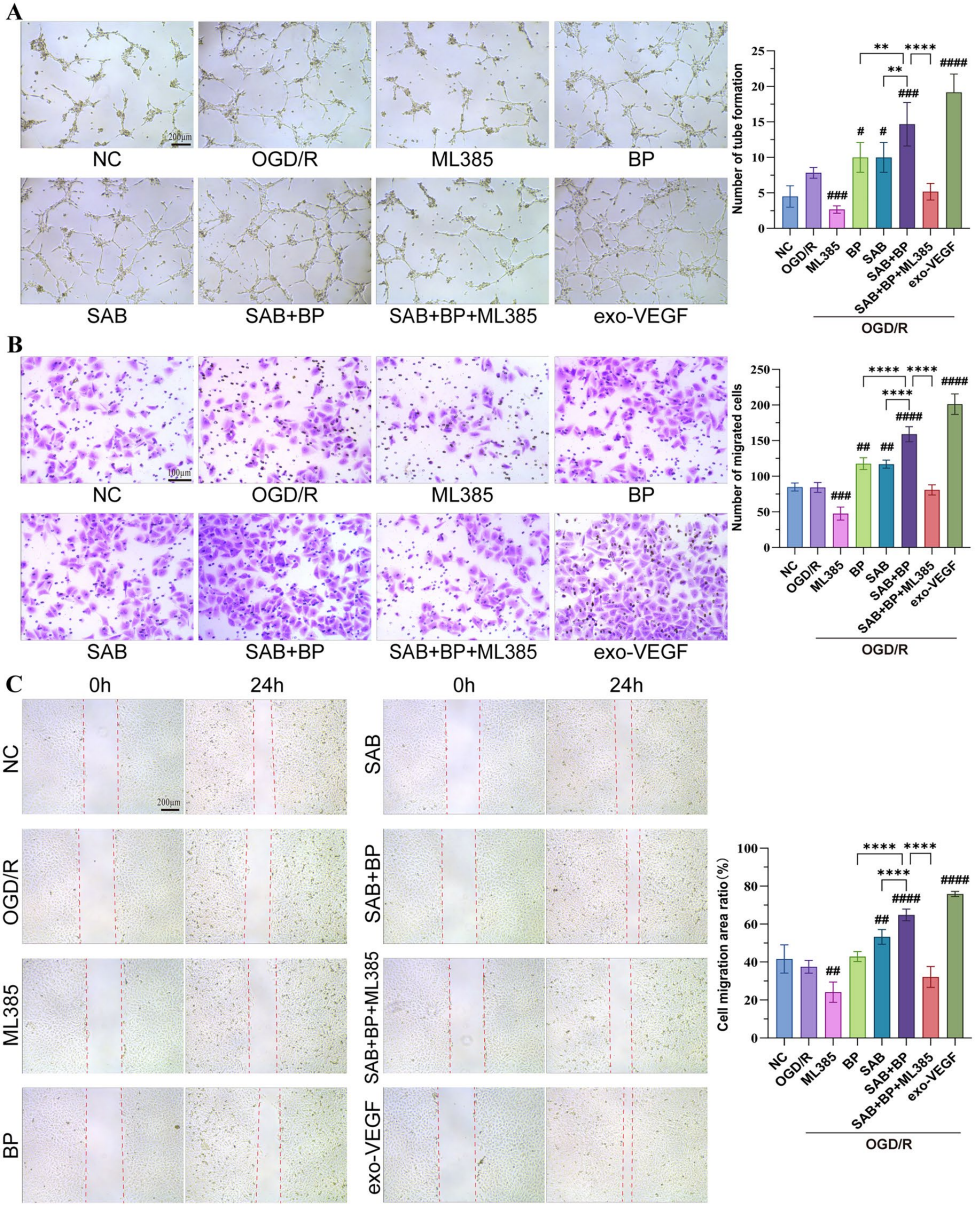
SAB-BP treatment promoted the vessel formation in HUVECs (*n* = 6–8). (A) Illustrative images and numerical analysis of the tube formation assay, (B) transwell assay, and (C) cell wound scratch assay (scale bar = 100 and 200 μm). Data are demonstrated as mean ± SD. Statistical significance: **p < 0.01, ***p < 0.001, and ****p < 0.0001 vs. SAB+BP group; #*p* < 0.05, ##*p* < 0.01, ###*p* < 0.001 vs. OGD/R group.

Cell viability significantly decreased after treatment with 0 − 80 µM SAB and 0 − 64 µM BP for 12, 24, and 48 h, exhibiting a dose- and time-dependent relationship. HUVECs treated with 20 µM SAB and 16 µM BP for 24 h were non-cytotoxic and showed increased cell survival rates. Thus, these concentrations were used in all subsequent *in vitro* experiments. We further evaluated the impact of SAB-BP on endothelial cell tube formation, wound healing, and transwell assays in HUVECs (Figure 6(A–C)). After OGD/R, the cell’s capacities to form tubule-like structures or to migrate after wounding were largely inhibited. All drug administration groups significantly enhanced cell tube formation, cell migration capacity, and scratch wound healing. In these assays, the combination of SAB and BP significantly improved the rescue effect compared to treatment with SAB or BP alone (*p* < 0.01, *p* < 0.0001). It was comparable to VEGF, used as the positive control. The Nrf2 inhibitor ML385 partially inhibits the enhancement of migration and tube formation in HUVECs induced by SAB-BP.

### SAB-BP activates Nrf2/HO-1/VEGFA signaling axis

**Figure 7.**
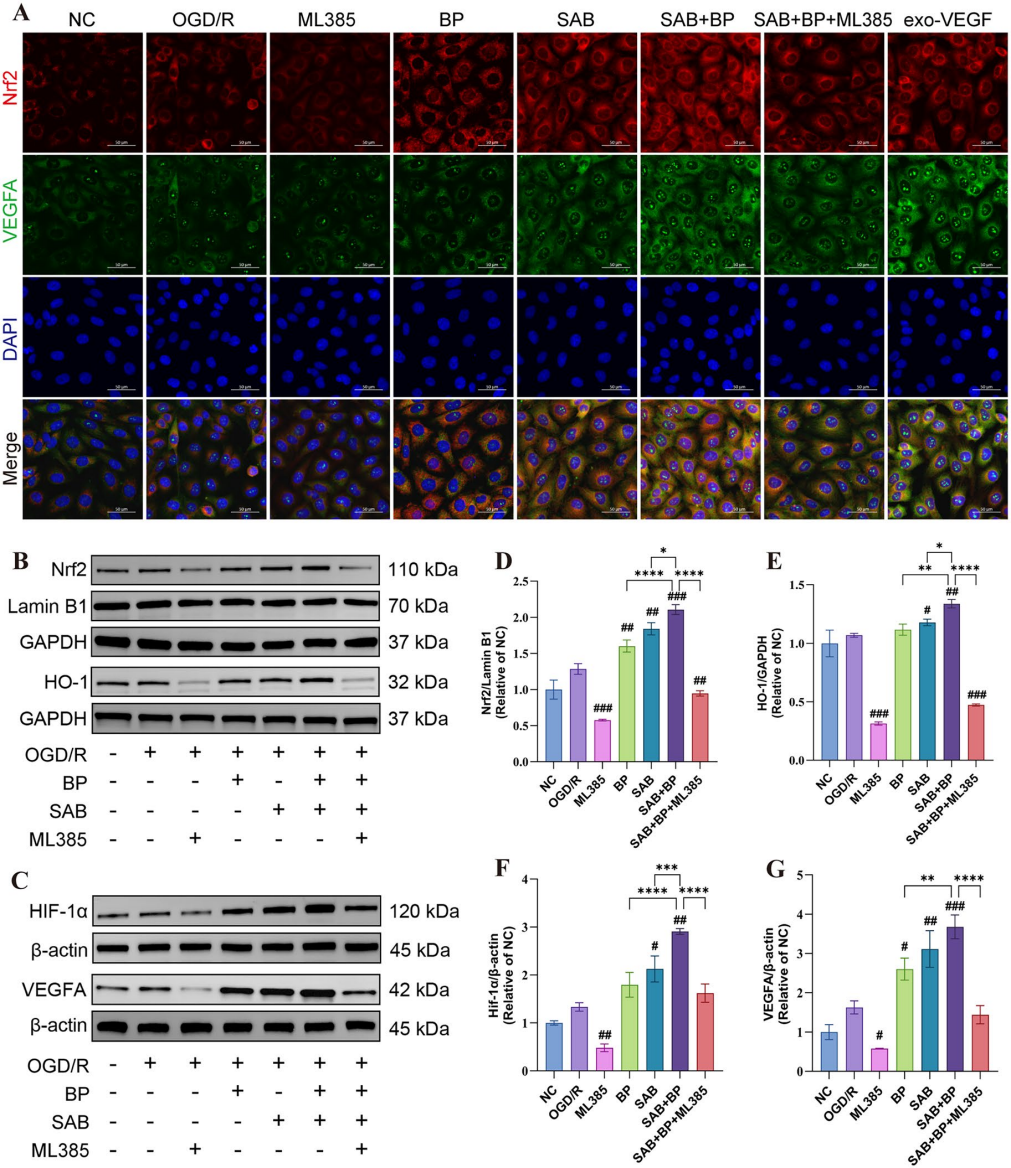
SAB-BP activates Nrf2/HO-1/VEGFA signaling pathways in HUVECs. (A) Immunofluorescent staining for VEGFA and Nrf2. (B, C) Western blotting of Nrf2, HO-1, HIF-1α and VEGFA levels. (D–G) Quantitative of the Western blotting in B and C. Data presented as mean ± SD (*n* = 3; *p < 0.05, **p < 0.01, ***p < 0.001, ****p < 0.0001 vs. SAB+BP group; #*p* < 0.05, ##*p* < 0.01, ###*p* < 0.001 vs. OGD/R group).

Numerous studies highlight the crucial involvement of Nrf2 in angiogenesis (Ji et al. 2014; Chen et al. 2019). The study further explored how SAB-BP promotes angiogenesis by upregulating VEGFA through the activation of Nrf2. The expression levels of Nrf2, HIF-1α, VEGFA, and HO-1 of different drug treatment groups in HUVECs were evaluated using immunofluorescence staining (Figure 7(A)) and western blot assay (Figure 7(B–G)). The drug treatment groups exhibited significantly elevated protein levels of Nrf2, HO-1, HIF-1α, and VEGFA compared to the OGD/R group. Additionally, protein expression significantly increased in the SAB-BP group when compared with the monotherapy group. Furthermore, we observed that the SAB-BP group may promote the proliferation of HUVECs when compared to the monotherapy groups. Our research demonstrates that ML385 treatment inhibited the SAB-BP-induced expression of Nrf2, HO-1, and VEGFA proteins, which facilitated angiogenesis and migration of HUVECs *in vitro*. Figure 7(A) illustrates that treatment with ML385 led to a simultaneous decrease in the fluorescence intensity of Nrf2, HO-1, and VEGFA. This implies that Nrf2 might occupy an upstream regulatory role.

## Discussion

Ischemic stroke is associated with blood stasis syndrome in both Western and Chinese Medicine, and therapies that enhance blood circulation and remove blood stasis may be effective (Greco et al. 2023; Liu et al. 2023b). The strong pro-angiogenic activity of Chinese herbal medicines that activate blood circulation and cease blood stasis offers a theoretical basis and potential for treating ischemic diseases. SAB, derived from Salvia miltiorrhiza, exhibits strong anti-oxidant, anti-inflammatory and pro-angiogenesis effects (Lv et al. 2015; Lin et al. 2018; Zhao et al. 2024b). BP, derived from Cortex Moutan, significantly suppresses the expression of major inflammatory mediators such as NF-κB and COX-1 (Kim et al. 2022). Based on TCM principles, various herbs with distinct properties can work together harmoniously to enhance therapeutic outcomes. Our preliminary research has demonstrated that the TCM herb pair ‘*Salvia miltiorrhiza* - *Paeonia suffruticosa* Andr.’ (Danshen-Mudanpi) holds therapeutic potential for ischemic stroke. Through *in vivo* component analysis, molecular docking, and molecular dynamics simulations, we further identified SAB and BP as the key pharmacologically active components underlying the efficacy of this herb pair in treating ischemic stroke (Zhao et al. 2024a). The potential for a combined SAB and BP therapy to enhance treatment efficacy for cerebral ischemia compared to individual treatments has not been investigated.

This study examined the effects of SAB combined with BP using MCAO and zebrafish models, as well as OGD/R-induced HUVECs. Clinical guidelines recommend that the time window for acute ischemic stroke thrombolytic therapy (e.g., rtPA) and thrombectomy is largely within 4.5 h of symptom onset (Meng et al. 2024; Zhou et al. 2025). This study initiates reperfusion 2 h post-ischemia to simulate the clinical scenario of ‘early intervention post-diagnosis’. Previous stroke model studies confirm SAB exerts maximal neuroprotective effects only with intervention before irreversible neuronal necrosis in early ischemic injury. Specifically, in a rat MCAO model, Fan et al. (Fan et al. 2018) showed immediate post-ischemia-reperfusion SAB administration significantly reduced cerebral infarct volume, alleviated edema, and mitigated inflammation. Likewise, Lv et al. (2015) demonstrated that such intervention improved rats’ neurological function, reduced infarct volume, and inhibited excessive astrocyte and microglia activation. Our investigations conclusively showed that the combined *in vivo* treatment of SAB and BP significantly decreased brain infarction volume and enhanced motor and somatosensory neurological function.

Through transcriptome sequencing, we detected extensive gene downregulation in the MCAO group, which is likely attributed to the ‘late recovery phase’ characteristics of cerebral ischemia. Brain tissue transitions from the acute response phase to tissue repair and neural remodeling stages at 14 days post-stroke. Genes associated with the acute ischemic phase (e.g., those regulating apoptosis, necrosis, excitotoxicity and inflammation) were highly expressed in the early stage. In contrast, as the acute phase resolves, these genes undergo prominent downregulation (Luo et al. 2022; Zhou et al. 2024). Transcriptome sequencing revealed that among the 297 overlapping DEGs, 46 were upregulated and 251 downregulated in the MCAO group vs. the SAB-BP group. SAB-BP may promote angiogenesis *via* regulating key genes including Nfe2l2, Vegfa, Vegfb, Fga, Mmp9, Thbs1, Serping1 and Tnfrs11a. Differential gene expression analysis revealed that these genes predominantly participate in essential signaling pathways, such as cytokine-cytokine receptor interaction, and complement-coagulation cascades. Western blot analysis and immunofluorescence staining indicated an upregulation of Nrf2, HIF-1α, and VEGFA proteins in the SAB-BP treatment group.

The zebrafish model has proven to be a practical and efficient tool for screening drugs targeting pro-angiogenesis or anti-angiogenesis in recent assays (Yu et al. 2013; Li et al. 2025a). The zebrafish embryo serves as an outstanding model for studying vascular development. The transparency of zebrafish embryos enables easy observation during development, and the *Tg(fli-1 EGFP)* transgenic line, which expresses fluorescent tags in endothelial cells, aids in studying blood vessel development. Our findings demonstrated that compared with SAB or BP monotherapy, the SAB-BP combination significantly reduced cerebral thrombosis formation and enhanced SIVs development in zebrafish, suggesting it exerts endothelial cell-protective and pro-angiogenic effects. Experiments conducted *in vitro* indicated that SAB-BP significantly promoted HUVEC tube formation and migration, with these effects being partially counteracted by an Nrf2 inhibitor. The SAB-BP combination demonstrated significantly greater therapeutic effects than administering SAB or BP alone. Molecular docking analyses were conducted between BP, SAB and Nrf2, VEGFA, respectively. The results demonstrated significant binding activity between BP/SAB and the target proteins, with binding affinities of −8.5, −8.4, −10.4 and −8.2 kcal/mol, respectively. The schematic diagram of molecular docking interactions is shown in Supplementary Figure 3.

Effective collateral circulation is vital during ischemic events, relying on angiogenesis, a complex process involving endothelial cell growth, sprouting, and the formation and branching of vascular structures (Hatakeyama et al. 2020; Fang et al. 2023). Nrf2, an essential transcription factor, alleviates oxidative stress by interacting with antioxidant response elements in the nucleus, thereby initiating the transcription of genes like HO-1, superoxide dismutase, and catalase. Recent research has extensively investigated the role of Nrf2 in promoting angiogenesis (Guo and Mo 2020). Nrf2 is vital for angiogenesis, potentially promoting vascular development by protecting the retina from oxidative stress caused by hyperoxia (Uno et al. 2010). Hypoxia can trigger the Nrf2-ARE pathways, leading to increased proliferation of abnormal tumor vessels. Inhibiting Nrf2 can potentially reduce tumor cell angiogenesis and migration (Liu et al. 2021; Xu et al. 2021). Research indicates that Nrf2 expression is upregulated and activated during vascular development, while its genetic deletion leads to decreased vascular density and impaired endothelial cell sprouting (Huang et al. 2018). Research indicates that hypoxia-responsive miR-101 and arsenic enhance Nrf2, HO-1, and VEGF proteins, aiding vascular tube formation and cell migration, whereas reducing Nrf2 significantly lowers HO-1 and VEGF levels (Meng et al. 2010; Kim et al. 2014). Our study shows that Nrf2 affects angiogenesis *via* the HO-1 mediated HIF-1α/VEGF pathway. Introducing the Nrf2 inhibitor ML385 to the SAB-BP group significantly reduced HO-1 and VEGFA protein levels. This suggests that SAB-BP may promote angiogenesis by activating the Nrf2/HO-1 pathway, as ML385 inhibits VEGFA overproduction. The pharmacological effects of BP may enhance SAB’s activity in the pro-angiogenic signaling pathway, or their combination may improve cellular absorption or metabolism. More investigation is necessary to understand the positive interaction mechanism between the two compounds.

Despite the intriguing findings obtained from this study, there are also some limitations in our work. Initially, we evaluated the impact of SAB-BP using zebrafish and rat *in vivo* models, as well as HUVECs *in vitro*. Future studies will utilize transgenic zebrafish lines to elucidate the roles of SAB-BP in promoting cerebral angiogenesis and protecting the blood-brain barrier. Second, this study lacked a dedicated assessment of SAB-BP’s impact on the proliferative capacity of HUVECs. Future research should conduct systematic evaluations using highly validated, well-established proliferation markers, specifically EdU and Ki-67. While pharmacological inhibition *via* ML385 robustly supports the regulatory role of SAB-BP in Nrf2 signaling, future studies incorporating genetic approaches (e.g., siRNA or CRISPR-Cas9) will further validate these mechanistic insights.

## Conclusions

**Figure 8.**
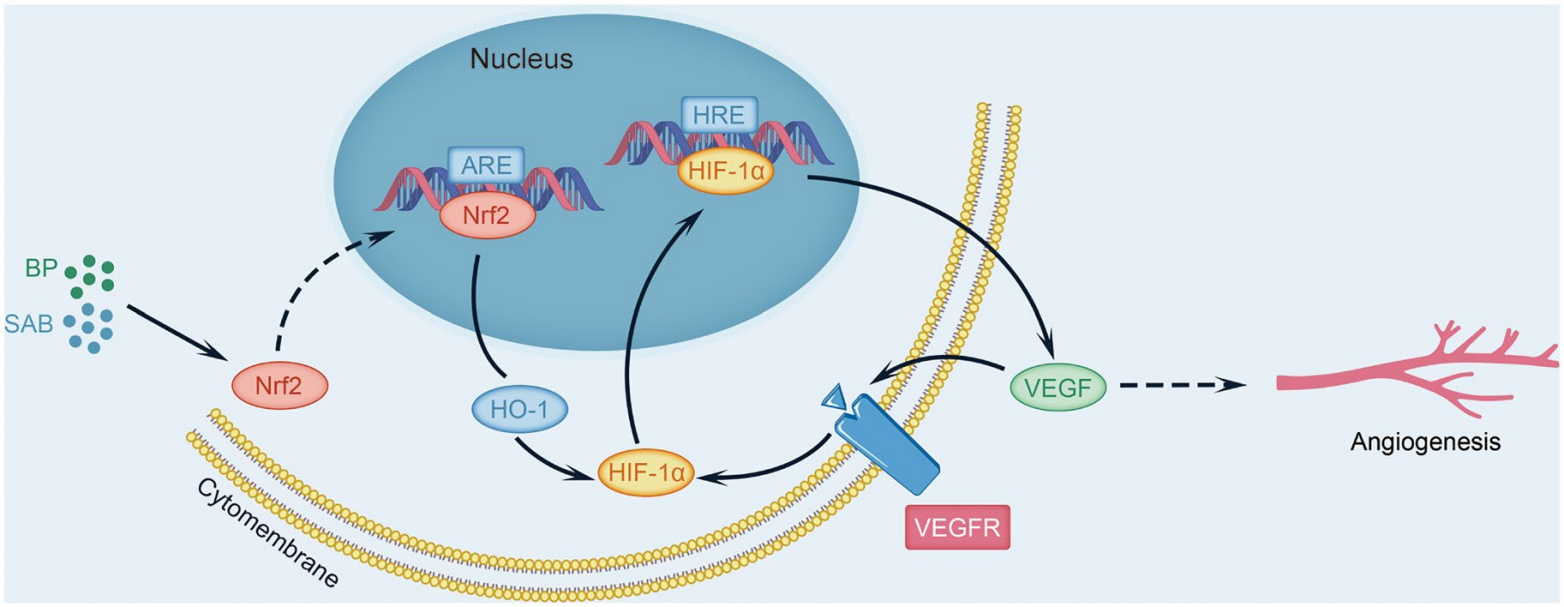
Diagram illustrating the mechanism through which SAB-BP enhances angiogenesis following ischemic stroke.

In summary, this research shows that SAB-BP treatment enhances angiogenesis in OGD/R-induced HUVECs, zebrafish, and MCAO rats by activating the Nrf2/HO-1/VEGFA signaling pathway, suggesting SAB-BP’s potential neuroprotective role in IS (Figure 8). This research offers novel insights into the pharmacological effects of SAB-BP for treating IS.

## Supplementary Material

Supplementary Table 1 revised.xlsx

Supplementary Figure 1 revised.tif

Supplementary Figure 3.tif

Supplementary Table 2.xlsx

Supplementary Figure 2.tif

ARRIVE Author Checklist.pdf

## Data Availability

Data will be made available on request. The rat transcriptome sequencing data in this study has been deposited in the SRA BioProject repository of NCBI, with the accession number PRJNA1297416. Corresponding author: Jingwen Wang, E-mail address: wangjingwen8021@163.com.
