# Supplementary figures and images for: The role of salvianolic acid B and benzoylpaeoniflorin in enhancing angiogenesis through Nrf2/HO-1/VEGFA signaling axis in ischemic stroke recovery
Source: Pharm Biol. 2025 Dec 23;64(1):67–86. doi: 10.1080/13880209.2025.2605571 (PMC12777785; doi:10.1080/13880209.2025.2605571)

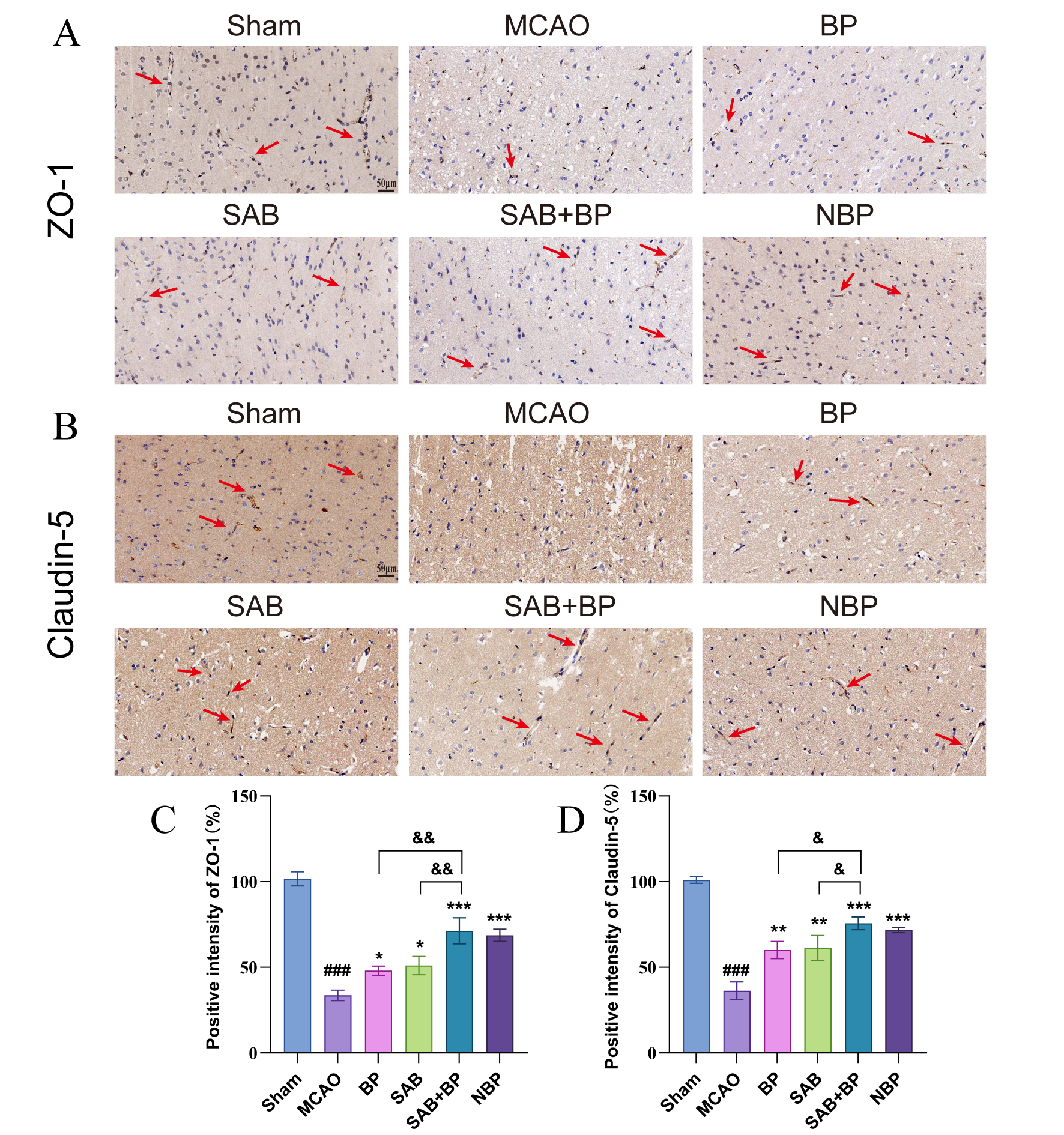

Supplement: Supplementary Figure 1 revised.tif [file IPHB_A_2605571_SM6077.tif]

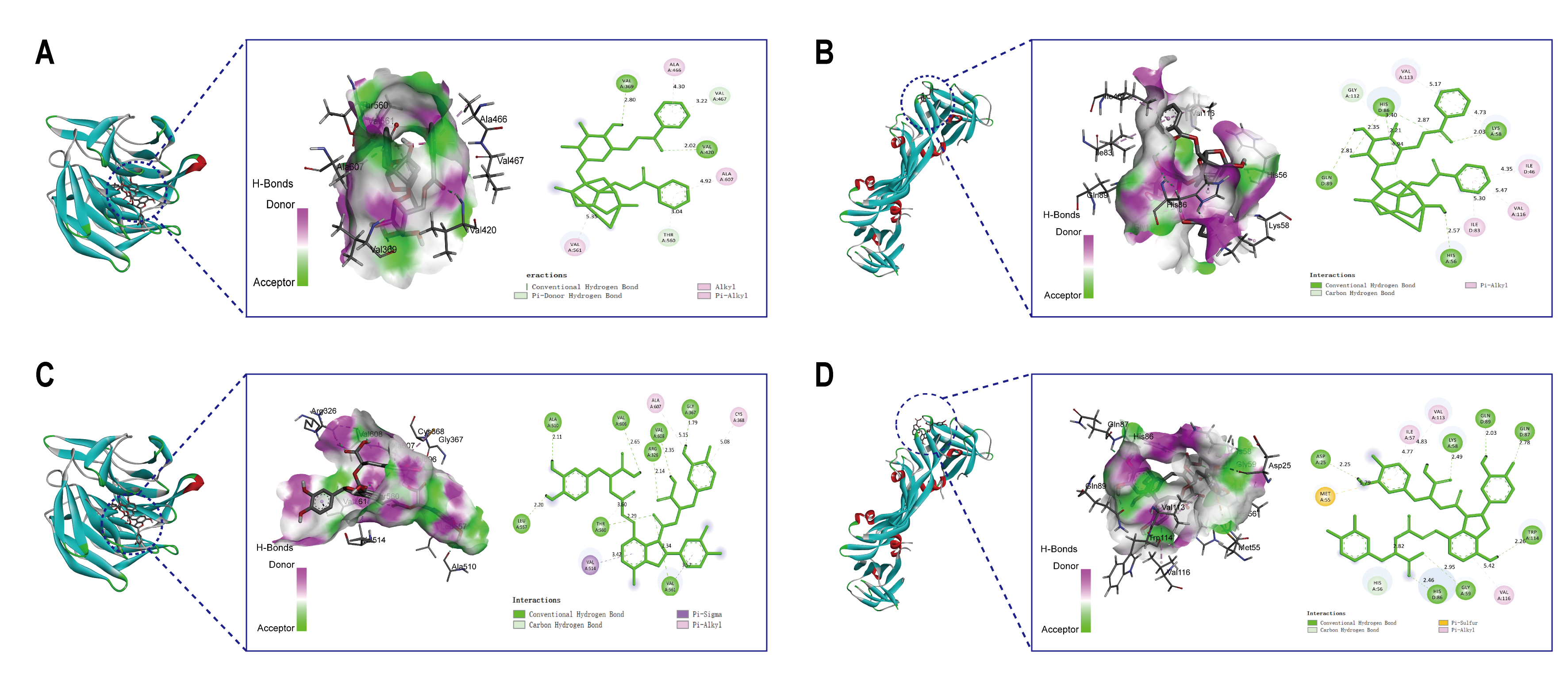

Supplement: Supplementary Figure 3.tif [file IPHB_A_2605571_SM6076.tif]

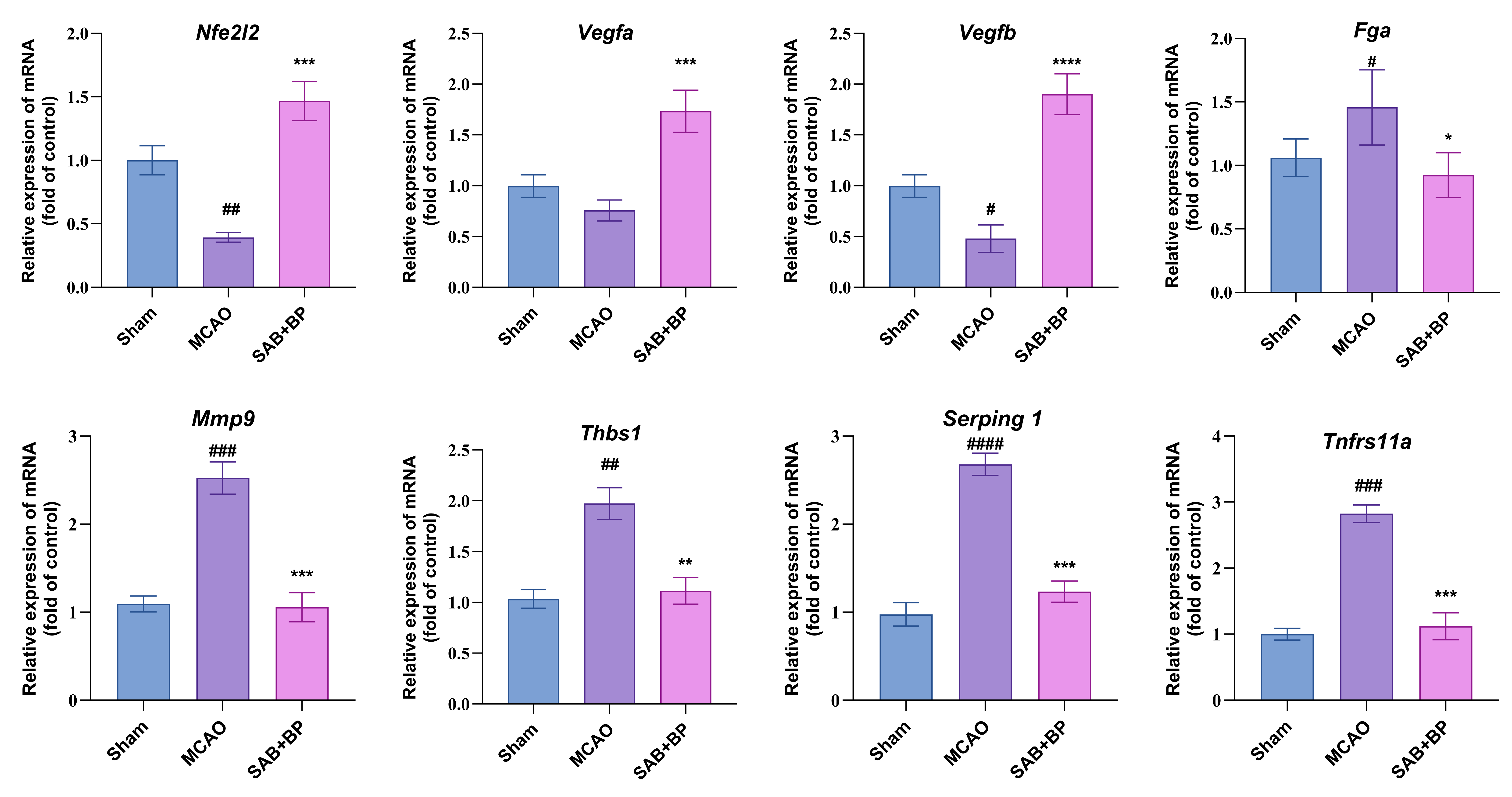

Supplement: Supplementary Figure 2.tif [file IPHB_A_2605571_SM6074.tif]
